# Clinicopathologic features of plasmablastic lymphoma: Single-center series of 8 cases from Saudi Arabia

**DOI:** 10.1186/s13000-015-0315-z

**Published:** 2015-06-25

**Authors:** Ghaleb Elyamany, Ali Matar Alzahrani, Muna Aljuboury, Najlah mogadem, Nagham Rehan, Omar Alsuhaibani, Abdulaziz Alabdulaaly, Eman Al-Mussaed, Imad Elhag, Abdullah AlFiaar

**Affiliations:** Department of Pathology and Laboratory Medicine, Prince Sultan Military Medical City, Riyadh, Saudi Arabia; Department of Hematology and Blood Bank, Theodor Bilharz Research Institute, Giza, Egypt; Department of Oncology, Prince Sultan Military Medical City, Riyadh, Saudi Arabia; Department of Basic Science, Princess Nourah Bint Abdulrahman, University, College of Medicine, Riyadh, Saudi Arabia

**Keywords:** Plasmablastic lymphoma, HIV, Chemotherapy, Outcome

## Abstract

**Background:**

Plasmablastic lymphoma (PBL) is a rare subtype of non-Hodgkin’s lymphoma. Characterized by its aggressive nature and plasmacytic differentiation, PBL remains a therapeutic and diagnostic challenge; it generally has a poor prognosis with very few long-term survivors and most patients dying within 2 years from initial presentation.

PBL has been reported in several other countries; however, there have been no reported cases from Saudi Arabia. Here, we report 8 cases of PBL depicting the clinical presentation, immunocompetency, immunphenotypic characterization, diagnostic challenges and treatment outcome.

**Methods:**

The medical records were reviewed for clinical presentation, staging, laboratory data, radiological studies, treatments, and outcomes. A broad immunohistochemical panel consisting of CD45, CD3, CD20, CD79a, Pax5, CD38, CD138, MUM1, EMA, Kappa, Lambda, CD 56**,** CD30, Bcl-2, Bcl-6, Alk-1, Ki-67, EBV-LMP-1, and HHV8 was performed.

**Results:**

The tumors predominantly exhibited immunoblastic/plasmablastic or plasmacytic morphologic features and had a plasma cell–like immunophenotype. All cases were immunoreactive for CD38, CD138 and MUM1 confirming plasma cell differentiation of the tumor cells. CD20 was negative for all cases; whereas CD79a and Pax5 were weakly positive in 2cases. All 8 cases were EBV-LMP-1/EBER-1 negative, and 1 case was HHV8 positive.

Similar to previously published studies, PBL in Saudi Arabia is characterized by male predominance (6/8), median age 51.5 years (mean age 46 years), associated with early dissemination, poor response to therapy, and limited survival (average survival time, 6.4 months, median overall survival 5.5 months). However, it does have some unique features. It occurs more commonly in immunocompetent persons (6/8, 75 %), is not associated with EBV infection (0/8), and nodal involvement (either primary or secondary) is common among patients (6/8).

In addition, extra-oral sites are more common than oral/nasal cavities (7/8) and the c-*myc* gene is not common (1/8, 12.5 %).

**Conclusion:**

It appears that PBL is heterogeneous in terms of clinical presentation and morphology. PBL is a therapeutic challenge with a clinical course that is characterized by its high rate of relapse and death. To date, treatment responses are usually partial and temporary. Therapies that are more intensive than CHOP do not seem to prolong survival. Further research is needed to understand the biology and molecular pathogenesis of PBL in order to improve therapies.

**Virtual slides:**

The virtual slides for this article can be found here: http://www.diagnosticpathology.diagnomx.eu/vs/1465801416161912

## Background

Plasmablastic lymphoma (PBL) is a rare aggressive subtype of non-Hodgkin’s lymphoma (NHL). Initially described in 1997, it occurs predominantly in HIV infected individuals and shows a predilection for the oral cavity [1–3].

Recently, dual infection with Epstein-Barr virus (EBV) and human herpesvirus 8 (HHV8) has been demonstrated in PBL. A number of cases have been reported in extra-oral sites, including the maxillary sinus, nasopharynx, stomach, small bowel, anus, lung, skin, soft tissues, heart and the spermatic cord [4].

PBL usually develops in middle-aged adults, with the age at onset ranging from 35 to 55 years [5], but it can occur in the pediatric age group [6, 7].

The clinical course of PBL is characteristically aggressive; the prognosis is poor, with death occurring between 1 and 24 months after diagnosis (average survival time, 6 months) [8]. Spontaneous regression has been reported with highly active antiretroviral therapy (HAART) [9–11] with a prolonged median overall survival (OS) time of 15 months [12] and durable responses to chemotherapy [13–16].

The clinical and histopathological features are frequently ambiguous, rendering the correct diagnosis quite difficult in the absence of an exhaustive integration of clinical, morphological, phenotypic and molecular features. The diagnosis of such neoplasms can be even more challenging in the setting of extra-oral localizations and in immunocompetent patients.

Here, we report 8 cases of PBL from Saudi Arabia depicting clinical presentation, immunocometency, immunophenotypic characterization and treatment outcome.

## Methods

### Study population

The study cohort comprised of 8 patients whose pathology slides were reviewed at Prince Sultan Military Medical City (PSMMC), Saudi Arabia between 2006 and 2014. All biopsies were obtained at diagnosis; all cases were reviewed for clinical data, primary site, staging, relevant laboratory data, radiological studies, treatment and outcome. This study was approved by the Ethical and Research Committee of the PSMMC.

### Morphological features

Hematoxylin-eosin (H&E) sections were reviewed, and morphological findings were summarized. The initial histological diagnosis was based on H&E staining and immunophenotyping results.

### Immunophenotyping

Immunohistochemical studies were performed on formalin-fixed paraffin-embedded tissue using an automated immunostainer and a broad panel of antibodies, including LCA, CD3, CD20, CD79a, PAX5, CD 56, CD10, Ki-67, HHV8, ALK-1, cyclin D1, CD38, CD138, Kappa and Lambda, CD30, bcl-2, bcl-6, MUM-1, EMA, HHV8 and EBV-LMP-1 according to the manufacturer’s instructions. Some markers were not assessed in all cases due to the limited amount of tissue.

### *In situ* hybridization for EBV

*In situ* hybridization (*ISH*) for EBV-encoded RNA (EBER-1) was performed on formalin-fixed paraffin-embedded tissue sections for 6 of the 8 cases with sufficient tissue using *ISH* according to the manufacturer’s instructions with the appropriate positive and negative controls. All cases were assessed for EBV-encoded LMP1 by immunohistochemistry (IHC).

### Cytogenetic analysis

Conventional chromosome analysis was performed on the other two cases by the standard cytogenetic protocols for neoplastic studies [17]. Fluorescent *in situ* hybridization (FISH) analysis was performed on all patients with the *MYC* probe (LSI *MYC* Dual Color, Break-Apart Rearrangement probe) to investigate the presence of *MYC* gene rearrangement. The FISH procedure was carried out on bone marrow aspirates (3 cases) and formalin-fixed, paraffin-embedded tissue sections (all cases), following the manufacturer’s guidelines.

### Statistical analysis

Clinicopathological variables were dichotomized to facilitate analysis and are presented using descriptive methods. Complete remission (CR) is defined as the disappearance of all clinical evidence of disease and the normalization of clinical symptoms, laboratory values, CT scans and a bone marrow (BM) biopsy (if initially involved) after treatment. The overall survival (OS) is defined as the time (in months) from diagnosis to the date of death from any cause or last follow-up.

## Results

### Clinical features

The clinical features of the patients are summarized in Table 1.

**Table 1:** Clinical features of PBL cases

Patient #	Case 1	Case 2	Case 3	Case 4	Case 5	Case 6	Case 7	Case 8
Age	59	58	52	20	33	43	52	51
Gender	Female	Male	Male	Male	Male	Male	Female	Male
Primary site (s)	Nasopharynx	Parotid mass/ Mandible	Iliac fossa mass	Skin/LNs	Neck masses /LNs	LNs	Ovary	Maxillary bone/tissue
Lymph nodes involvement	No	Yes	Yes	Yes	Yes	Yes	Yes	No
Other Organs involved	CNS	Spleen	Lungs	CNS	Spleen	Spleen	GIT	No
	Multiple bone	Liver		Liver	Liver	Liver		
		Lungs				Lungs		
B-symptoms	Yes	Yes	No	Yes	Yes	Yes	Yes	No
HIV status	─	─	─	─	─	+	─	+
BM involvement	+	─	+	+	─	+	+	ND
Stage	IV	IV	IV	IV	IV	IV	IV	I
Lytic lesions	Yes	No	No	No	No	Yes	Yes	No
M protein	IgG	─		IgM	─	─	─	─
CSF cytology	─	─	─	─	─	─	─	─
Therapy	CHOP-R	ESHAP	CHOP-R	Bortezomib	ESHAP	Palliative	Palliative	CHOP-R
	Radiotherapy			Dexamethasone				
Response to chemotherapy	Partial	No	Partial	No	Partial	N/A	N/A	Partial
Outcome	Died of disease	Died of disease	Died of disease	Died of disease	Died of disease	Died of disease	Died of disease	Died of disease
Survival time (months)	12	3	9	1	7	2	4	13

*ND* Not done, *N/A* Not applicable

Of the 8 cases, 6 were males and 2 were females with an age range of 20–59 years (median age 51.5 years). Seven patients were categorized intermediate- to high-risk IPI and one case was low risk. Two of the patients (25 % - cases 6 and 8) were positive for HIV, and 1 patient was infected with human herpes virus 8 (HHV8 - case 2).

The primary involved locations were lymph nodes, the parotid gland, skin, nasopharynx, ovary, maxillary bone and iliac fossa. Seven cases presented with disseminated disease stage IV, multifocal nodal involvement (6 cases), and an extra-nodal disease involving the liver, spleen, soft tissue and CNS.

Of the 8 patients, four had some clinical features of plasma cell myeloma, which includes monoclonal serum immunoglobulin spikes and/or lytic lesion; however, all four patients had normal serum calcium, blood urea nitrogen, and creatinine. Lytic bone lesions were observed in three patients. Two patients had monoclonal serum immunoglobulin spikes (case 1 and case 7). The monoclonal immunoglobulins were IgG and IgA. The patient with IgG had multiple lytic bone lesions determined by computed tomography (CT) scans. The other patient had a monoclonal IgA spike, but there was no evidence of lytic bone lesions.

Three of our PBL cases were treated by CHOH-R and had an average OS of 11 months. Two of our cases received a combination of chemotherapeutic drugs including etoposide, methylprednisolone, high-dose cytarabine and cisplatin, (ESHAP) and had an average OS of 5 months. One of the cases (case 4) was treated by bortezomib with dexamethasone and died after 1 month of final diagnosis due to disease progression. Two of the patients received palliative chemotherapy due to the patients’ clinical condition and had a mean survival of 3 months. Intrathecal chemotherapy was administered to patients with CNS involvement or patients at risk of CNS involvement.

### Pathologic features

#### Histologic findings

**Fig. 1:**
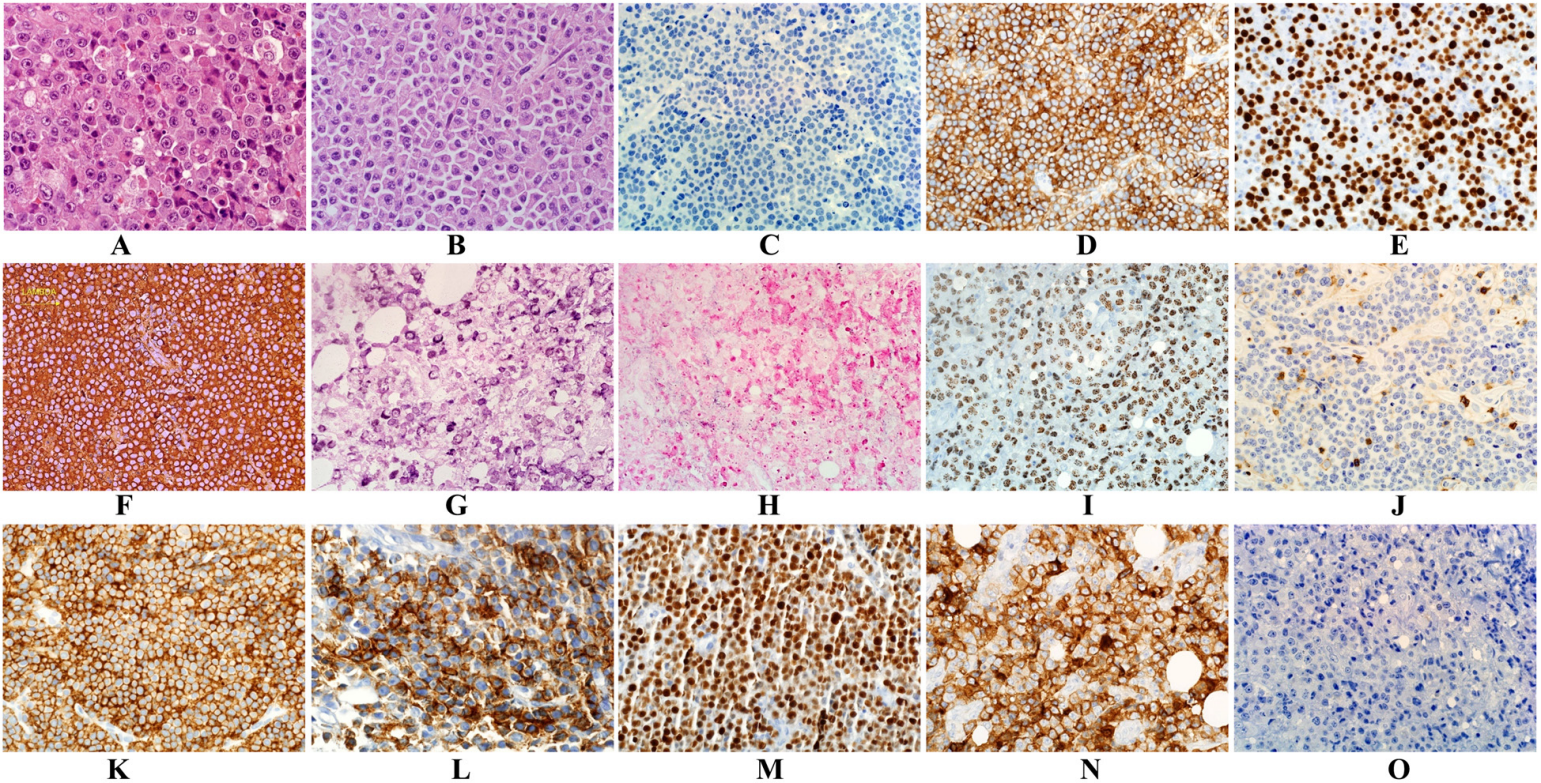
**a**–**o**: Histomorphologic features and phenotypic profile of PBL cases. **a** & **b** H&E, PBL consists of a population of large lymphoid cells showing plasmablastic/immunoblastic morphology (**a**), cases 2 or plasmacytic morphology (**b**), case 7. **c** & **d** PBL typically lacks CD20 expression (**c**) and is strongly positive for CD138 (**d**), case 1. **e** Staining with Ki-67 shows a high proliferation rate, case 7. **f **& **g** Lambda, light chain shows positivity in neoplastic cells by immunohistological stain, case 4 and by ISH respectively, case 2. **h** &** i** Tumor cells show negativity to EBER *in situ* hybridization (**h**) and positivity to HHV8 by immunohistological stain (**i**) respectively, case 2. **j** and **k** PBL shows negativity to CD45, case 8 whereas shows strong and diffuse positivity in case 4 (**k**). **l** & **m** The tumor cells are positive for EMA (**l**), case 4 and positive for MUM-1 stain in case 7 (**m**). **n** & **o** Case 2 shows strong positivity for CD30 staining (**n**) and negative for ALK-1 immunohistochemical staining (**o**)

All PBL cases showed morphologic features of diffuse proliferation of predominantly large lymphoid cells with plasmablastic, immunoblastic or plasmacytic morphology. The number of the more mature plasma cells ranged from minimal to moderate in the various cases. The neoplastic cells had round nuclei, vesicular chromatin, smooth nuclear contours, prominent central/peripheral/or multiple small nucleoli, variably eccentrically located nuclei, and abundant cytoplasm (Fig. 1).

The bone marrow was diffusely infiltrated by large cells in 5 cases with morphological features similar to tissue sections. All CSF cytology was negative for malignant cells even though radiological work up showed evidence of CNS involvement in 2 cases.

### Immunophenotype

The IHC characteristics are summarized in Table 2. The tumor cells of PBL exhibit features of terminally differentiated B-cells and express antigens in a pattern similar to plasma cells. All cases were positive for CD38, CD138, and MUM1; variably positive for CD45, CD79a, Pax5, EMA and CD30. All cases were nonreactive to CD20, and one case (case 1) was positive for CD56. The proliferation index showed a high range from 70 to 90 % (the average was 76 %). EBV infection was negative in all 8 cases examined (EBV positivity was determined on the basis of EBER-1 *in situ* hybridization (6/8) and immunohistochemical staining of EBV-LMP1 (8/8); whereas HHV 8 was positive in one case (case 2). Only one case was positive for C-*MYC* gene by IHC (Fig. 1).

**Table 2:** The immunohistochemical characteristics of the PBL cases

Pt.#	CD45	CD20	CD79a	PAX5	CD3	CD38	CD138	MUM1	EMA	CD56	KAPPA/ LAMBDA	ALK1	CYCLIN D1	EBV- LMP1	HHV8	CD10 BCL2 BCL6	Ki-67
Case 1	─	─	─	─	─	+	+	+	─	+	Lambda	─	─	─	─	─	>90 %
Case 2	+	─	─	─	─	+	+	+	+	─	Lambda	─	─	─	+	─	>80 %
Case 3	─	─	─	─	─	+	+	+	─	─	ND	─	─	─	─	─	>80 %
Case 4	+	─	─	+/─	─	+	+	+	+	─	Lambda	─	─	─	─	─	>80 %
Case 5	+/─	─	─	─	─	+	+	+	+	─	ND	─	─	─	─	─	>70 %
Case 6	─	─	+/─	─	─	+	+	+	─	─	Lambda	─	─	─	─	─	>70 %
Case 7	─	─	+/─	+/─	─	+	+	+	─	─	Kappa	─	─	─	─	─	>70 %
Case 8	─	─	─	─	─	+	+	+	+	─	Kappa	─	─	─	─	─	>70 %

*ND* Not done

+/−: Weak positive, +: Positive, −: Negative

### Cytogenetic analysis

Because conventional chromosome analysis only was obtainable in two cases, correlation with conventional karyotypic analysis was not possible.

**Fig. 2:**
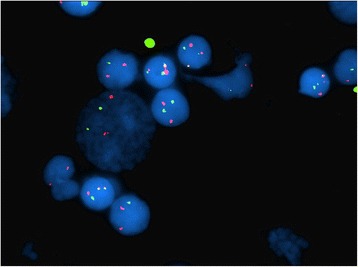
FISH study performed on interphase cells using probes to the MYC (red signal) and IGH (green signal) loci demonstrates that the neoplastic cells contain a reciprocal translocation between the MYC and IGH loci (yellow signals), case 7

All but one case (case 7) showed no presence of MYC translation after going through FISH analysis Fig. 2. One case (case 1), showed complex cytogenetic changes that are more commonly observed in plasma cell myeloma with extra copies of chromosomes 8, 11, and 17 (40 %) indicating the presence of a hyperploid tumoral clone and was reported as nuc ish (TP53 × 3)[80/200]/(CCND1) × 3 (IGH) × 2 [80/200]/(5′MYC, 3′MYC,5′MYC con 3′MYC) × 3[80/200].

## Discussion

PBL is a rare aggressive B-cell NHL that is often related to EBV infection; it accounts for 2.6 % of all AIDS-related lymphomas with overall survival rates between 6 and 12 months [18, 19]. The available pathological, immunohistochemical, molecular, and genetic data suggests that plasmablastic lymphoma arises from post-germinal centre, terminally differentiated, activated B-cells that are in transition from immunoblasts to plasma cells [20]. Array comparative genomic hybridization (involving 16 cases of PBLs) demonstrates that despite the high degree of immunophenotypical similarity between PBL and plasma cell myeloma (PCM) [21], the genomic aberration pattern of PBL is more similar to DLBCL than to PCM [22].

Similar to previously published studies [3], our patients are characterized by male predominance (6/8). Though the reason for this observation is not clear, it may be due, in part, to the over-representation of HIV infection in men compared to women in developed countries [23]. Another contributing factor could be the fact that a number of cancer-related genes, known as cancer/testis antigens (CTAs) that are located on the X-chromosome (MAGEA1, SSX1, SSX4, and CTAG1/2) [24–26], are highly upregulated in PBL, which mainly affect male individuals [27]. However, CTAs can be aberrantly expressed in different types of cancer [28, 29].

In PBL, extranodal involvement occurs much more frequently than nodal involvement with a predilection for the oral/nasal cavity [12]. In this study, nodal involvement (either primary or secondary) is common among patients. (6/8) However, extra-oral sites were more common than oral sites. These differences may be attributed to the small sample size.

EBV has been found to be associated with AIDS related lymphomas and is thought to play a key role in its pathogenesis. In one study, there was a 70 % association between PBL and EBV [12, 30–32]. As reported by Delecluse *et al.*, [1], 9 of 15 cases were found to be associated with EBV using *ISH*; of these 9 cases, 5 demonstrated expression of latent membrane proteins (LMP-1). In another study of oral cavity PBL, 10 of 12 cases tested positive using EBV-EBER *in situ* hybridization, but none of the 12 were reactive for EBV-L MP-1 [13, 33]. In our study, EBV-LMP-1 also failed to react in all 8 cases examined. Although this technique is less sensitive than *ISH*, our study failed to detect the presence of EBV in all 8 cases. Six cases tested by EBER-1, were non- reactive for EBV, thus conflicting with previous reports that demonstrated a strong association and a role of EBV infection in tumorigenesis in a portion of PBL cases [1, 12, 33]. However, this does not hold true for all cases of PBL, as demonstrated in our study and other reported cases of EBV-negative PBLs [1, 33–35]. The significant value of association between EBV expression and PBL cases is unclear. Some studies have shown a better outcome in immunocompetent patients with PBL, while others have shown that EBV expression is not associated with the outcome of HIV-associated PBL [36–38]. In brief, our results (though the sample size is small) shows that EBV infection is not the sole cause of the disease.

Although PBL was originally associated with AIDS, an increasing number of reports has described cases arising in immunocompetent individuals [2, 36, 39]. It is currently unclear if HIV status alone confers a better prognosis in patients with PBL. In a recent review of literature, patients with PBL and HIV infection were found to have an OS of 14 months compared to 9 months in HIV-negative patients [36]. A review of 112 HIV positive patients with PBL showed a median overall survival (OS) of 15 months and a 3-year OS rate of 25 % [12], compared to a review of 76 HIV-negative PBL patients that showed a median OS of 9 months with a 2-year OS rate of 10 % [40].

The reasons for the better outcome are unclear, but a potential explanation for this finding is that the use of highly active antiretroviral therapy (HAART) may restore immune surveillance to combat the tumor more efficiently. However, other studies do not support this result [27, 36, 41].

In our PBL study, 2 male patients of the 8 cases (25 %) were positive for HIV and had an average OS of 6.5 months which is similar to HIV negative patients (6 months). This may be attributed to the fact that our patients were not on HAART at presentation and the small sample size.

PBL is associated with rearrangements in the *c-* MYC gene in more than 40 % of cases [42, 43]. Valera and colleagues reported *c-* MYC rearrangement in 50 % of the cases reviewed and noted that EBV positive patients are more likely to have the *c-* MYC abnormality and *c-myc* rearrangement occurs less frequently in EBV negative patients [44]. The presence of a MYC/IgH rearrangement is associated with a worse overall survival [38, 42, 44]. In our study, the c- MYC gene is not common among our PBL series as FISH analysis failed to detect the *presence* of *MYC* translocation in 7 patients and only one case (case 7) was positive for *MYC* translocation. The discrepancy between our results and those previously reported may be due to a small sample size, ethnic characteristics, or molecular characteristics of this lymphoma in our population. In addition, all cases were EBV negative and may serve as a contributing factor since c-myc rearrangement occurs less frequently in EBV negative patients as reported by previous studies [44].

Since there is no standard chemotherapy protocol for treatment of this type of lymphoma, 3 of our PBL cases were treated by anthracycline based protocols (Cyclophosphamide, Adriamycin, Vincristine, Prednisolone and Rituximab or CHOP-R), and 2 cases received a regimen combination of chemotherapeutic drugs including etoposide, methylprednisolone (solumedrol), high-dose cytarabine (ara-C) and cisplatin (ESHAP). Both of these regimens were combined with radiotherapy when indicated. Two cases received palliative chemotherapy (due to the patients’ clinical condition), and one case was treated by bortezomib which is a proteasome inhibitor and a cornerstone in myelomas therapy and relapsed or refractory mantle cell lymphoma [45]. Although CD20 expression was nonreactive in all cases, we used anti-CD20 monoclonal antibody rituximab with CHOP protocol, though its use in such cases are of uncertain utility [46].

Among the 6 patients who received chemotherapeutic regimens, none of them (0 %) achieved complete remission (CR), 4 (66 %) had a partial response (PR) and 2 (33 %) did not respond (NR) or progress. Since the sample size is small, we cannot give conclusive results, but it appears that the more intensive regimen ESHAP does not show a survival advantage when compared to the less intensive CHOP-R regimen. The treatment responses were partial and temporary in both regimens which is similar to the results that have been published in several past studies [38, 47]. Patients with PBL that were not treated with chemotherapy invariably died with a median survival of 3 months [36]. Similarly, 2 of our patients received palliative therapy and died with a median survival of 3 months. The patient (case 4) that was treated with bortezomib, a proteasome inhibitor with dexamethasone, was without improvement and died after one month of final diagnosis due to disease progression.

Generally, PBL has a poor prognosis with very few long term survivors and most patients dying within 2 years from initial presentation [48]. The survival outcomes in this study are similar to those published in other studies [12, 49]. The median OS was 5.5 months (average survival time 6.4 months) from diagnosis but varied considerably (range, 1 to 13 months). The average survival time reported by Delecluse *et al.* [1], was a few months, and half of the 16 patients with available follow up, died within 12 months of diagnosis. In one series of 13 patients, all died within 34 months with a median survival of 7 months. [11] Of the 90 cases in literature that mention survival data, almost half (47 %) died within 1 year of diagnosis; others report a very short survival [11, 35].

More recent reports have reported improved survival in HIV infected PBLs when treated with both HAART and appropriate chemotherapy. This is similar to outcomes of HIV infected patients with other NHLs [7, 12]. However, the outcome presented here is inferior to that recently reported [7, 12, 16]. The difference may be attributed to several factors, including ethnic and molecular differences. Most of the cases (7/8, 95 %) presented with Ann Arbor stage IV and 6/8 are HIV negative which are associated with poor prognosis [21, 26].

Because the clinical and histopathological features are usually ambiguous, PBL remains a diagnostic challenge. Rendering the correct diagnosis can be quite difficult in the absence of an exhaustive integration of clinical, morphological, phenotypic and molecular features. The diagnosis of such neoplasms can be even more challenging in the setting of extra-oral localizations and in immunocompetent patients. The differential diagnosis with the activated B-cell-like (ABC-like) subgroup of DLBCL and PCM with plasmablastic morphology is still a common problem due to the lack of a distinctive phenotype. The differential diagnosis includes immunoblastic DLBCL and other lymphoid neoplasms with plasmacytic features such as ALK-positive DLBCL, primary effusion lymphoma (PEL), BL with plasmacytoid differentiation and plasmablastic plasmacytoma/myeloma (Table 3). Immunoblastic DLBCL and BL can be excluded on the basis of the characteristics CD20 and LCA positivity in combination with negative markers of plasma cells, such as CD138 [1, 8]. PBL is distinguished from ALK-positive DLBCL by its lack of expression of the ALK protein, and the absence of HHV8 co-infection distinguishes PBL from PEL which usually manifests as pleural or pericardial effusion and rarely associates with lymphadenopathy or mass. PBL is also variably positive for CD30, epithelial and endothelial markers such EMA and CD31, posing some problems in differential diagnosis with poorly differentiated solid tumors [50].

**Table 3:** Differentiating PBL from other Neoplasms by Immunophenotyping

Parameter	PBL	Burkitt	Anaplastic DLBCL	PEL	ALK + DLBCL	PCM/Plasmacytoma	PLASMBLASTIC PCM/Plasmacytoma
Clinical presentation	frequently oral cavity	Often extranodal (jaws and orbits)	Wide variety of presentations	Involves body cavity	Wide variety of presentations	BM (Extramedulary in plasmacytoma)	BM (Extramedulary in plasmacytoma)
Immunocompetency	+/−	++	+++	+/−	+	++	++
Association with HIV	+++	++	++	+++	-	-	-
Association with HHV8	+/− (usually -)	-	-	+	-	-	-
LCA	+/−	+	+	+/−	+/−	+/−	+/−
B-Cell Markers							
CD20	-	+	+	+/−	-	-	-
CD79a	+/−(usually -)	+	+	-	-	+/− (usually -)	+/− (usually -)
CD138	+	-	-	+	-	+	+
CD56	+/−(usually -)	Rare +	Rare +	Rare +	+/−	Usually +	Usually +
Ki67	High >70 %	High >90 %	High <90 %	High >80 %	High >80 %	Low	High >70 %
Other	BLIMP1+	CD10 +	BCL-6 Usually +	CD30 Usually +	ALK+	Serum M-spike CRAB	Serum M-spike CRAB

CRAB: hypercalcemia, kidney disease, anemia, and bone lytic lesions

Similar to previously reported studies, the main differential diagnosis in our study was plasmablastic PCM particularly if extramedullary. Even though it is difficult, it is clinically important and critical to differentiate between these two entities, as treatment for the twodiseases is significantly different [21].

In practice, morphologic distinction is not always possible. PBL, particularly when associated with paraproteinemia and bone lesions, (as in case 1, 3 and 4) is very difficult to distinguish from PCM with transformation. The designation of these neoplasms is based mainly on clinical presentation like de novo presentation as aggressive tumors with plasmablastic morphology, predominantly extramedullary, often with mucosal involvement, and associated with HIV and EBV. These characteristics favor PBL. PCM rarely present de novo as high-grade lesions and the association with EBV and HIV, although previously reported, remains quite rare [51, 52]. Recently, an immunohistochemistry stain for BLIMP1 and XBP1; markers of terminal B-cell differentiation have been proposed to identify PBL; however, this finding remains investigational [53] and these markers are often not routinely available. There is some controversy whether PCM and PBL represent two separate entities or are part of a common pathway. Moreover, Qing and colleagues reported a case where PBL occurred as a result of transformation from a plasmacytoma [54]. However, the distinction between the two has important clinical and therapeutic implications.

## Conclusion

PBL has a predilection for immunocompromised individuals based on its prevalence in both HIV-positive patients and in those undergoing solid organ transplantation. PBL may be poorly recognized by pathologists, which is partly attributable to its relatively rare occurrence and unusual immunophenotype. Morphologically and genomically speaking, PBL is best classified as a form of DLBCL. However, based on immunohistochemical data, PBL is much more similar to an extramedullary PCM. Clinically, it has a predilection for oral cavity involvement, a feature not typically seen in other lymphoid malignancies. Recent studies have shown a high prevalence of MYC translocations that may contribute to its aggressive nature. The pathogenesis has not been clearly defined but is thought to involve dysregulation of terminal B-cell differentiation and apoptosis, potentially due to the effects of MYC translocation and EBV infection. PBL is a therapeutic challenge with a clinical course characterized by a high rate of relapse and death. Overall, PBL is associated with early dissemination, poor response to therapy, and limited survival. To date, treatment responses are usually partial and temporary. Treatment has been centered on CHOP chemotherapy with a good initial response; however, due to a poor survival rate, more intensive therapies than CHOP or CHOP-like have been recommended but do not seem to prolong survival. Recent case reports a good clinical response to bortezomib which is encouraging and may provide clues to the underlying pathophysiology of PBL. Finally, it is worthwhile to highlight that no prospective therapeutic trials have been done specifically in patients with PBL. In order to improve therapies, further research is needed to understand the biology and molecular pathogenesis of PBL, which seems to be very heterogeneous. To date, the survival outcome for PBL remains very poor regardless of intervention.

## Abbreviations

CHOP: Cyclophosphamide, adriamycin, vincristine and prednisone
CHOP-R: Cyclophosphamide, adriamycin, vincristine, prednisone and rituximab
ESHAP: Etoposide, methylprednisolone (solumedrol), high-dose cytarabine (ara-C), and cisplatin
IPI: International Prognostic Index
OS: Overall surviva
HAART: Highly active antiretroviral therapy
PBL: Plasmablastic lymphoma
HIV: Human Immunodeficiency Virus
PSMMC: Prince Sultan Military Medical City
